# On the Present State of the Evidence with Regard to the Vaccine Inoculation

**Published:** 1800-05

**Authors:** George Pearson

**Affiliations:** Physician to St. George's Hospital, &c.


### *On the present State of the Evidence with regard to the Vaccine Inoculation.*

*By* GEORGE PEARSON, M. D. F. R. S. &c. *Physician to St. George's Hospital, &c.*

IN the last Number of the Medical and Physical Journal, Dr. Denman appears to have undertaken the task of giving a view of the principal facts concerning the Cow-pock, to estimate the value of the new Inoculation. Although that statement, if examined strictly, according to the best evidence, would be found incorrect in several points, I do not think it necessary to propose any alteration; but as many facts are omitted, which throw light on the nature of the Cow-pock disease, and afford strong recommendations of the Inoculation, I am inclined to believe that the propriety of some additions will be admitted. It is but justice to observe, that as no one can doubt the purity of the motives of the respectable writer of the above article, the partiality of his statement, (if it be a partial one) I humbly presume, can only be owing to his not having had opportunities of better information.

In the months of January and February, 1799, the breaking out of the Cow-pock in two milk farms near London, furnished the Vaccine poison which Dr. Woodville and myself disseminated, not only throughout this island, but also over a great part of the continent, as well as to other quarters of the globe; and by which not fewer than five thousand persons, at least, may be counted, who have undergone the Cow-pock Inoculation. This experience has taught us, among other new facts:

1. That no serious consequences are to be apprehended from the inflammation and ulceration of the inoculated part; where-

 as, as, previouſly to the above date, (January and February, 1799) thoſe who were conſidered to have had the moſt experience in the inoculation of the Cow-pock, were themſelves apprehenſive of ſuch conſequences, *particularly with reſpect to infants.* Accordingly, in the ſpring of 1799, the firſt trials in theſe tender ſubjects were not made without the moſt painful anxiety.

2. For the inflammation of the inoculated parts neither *cauſtics* nor *eſcharotics* have been found neceſſary, in the hands of thoſe who have had by far the moſt extenſive experience. In fact, topical applications have rarely been wanted; and when they have, the uſual ſedative ones for inflammations were found efficacious.

3. The ſlightneſs of the diſeaſe from the vaccine inoculation is now aſcertained, as well as its ſafety, from the reports of many practitioners; and as it does not appear that more than one out of 6000 has died under the new practice, it ſeems not unreaſonable to infer, that the chance of life is, perhaps, greater during the inoculation, than under the ordinary circumſtances to which human creatures are expoſed. The ſafety of the practice is confirmed by the inoculation ſo long practiſed among the peaſants in ſeveral parts of England, in the rudeſt manner, with pointed knives, awls, &c. time immemorial.

4. The permanent nature of the vaccine poiſon appears now fully determined; for it ſeems fair to calculate, that at leaſt 5000 perſons have been inoculated with the matter originally taken in January and February, 1799, by Dr. Woodville and myſelf, from the cows in Gray's Inn Lane; and by myſelf, excluſively, from Mr. Willan's cows, in Marybone Fields; and yet the characters of the inoculated Cow-pock are the ſame now that they were in the firſt inſtances directly from the animal.

5. With a liberality above moſt men, and with a zeal which has rarely been equalled, Dr. Woodville has conducted the practice of inoculation, both of the vaccine and variolous matter, in the ſame, and in different ſubjects, by which ſome very curious facts, relating to the agency of both poiſons, have been diſcovered.

6. The Cow-pock matter of the human animal has been ſhewn to be capable of producing the ſame diſeaſe by inoculation of cows, as that which is produced in cows by their own ſpecies.

7. Two kinds of eruptions have been obſerved to occur from the vaccine inoculation, viz. one kind, probably the ſame as the Small-pox, or, at leaſt, ſcarcely diſtinguiſhable from the Small-pox, owing perhaps to the agency of the variolous poiſon at the ſame time with the vaccine; the other kind of eruptions alſo reſemble the variolous in ſeveral properties, but differ fer from them in the time of their appearance, and in their duration; which have occurred in the practice of almost all extensive inoculators, although every imaginable precaution has been taken to avoid the introduction of variolous matter.*

8. Direct experiments of inoculation with the matter of grease (from horse's heels), as well as a great mass of circumstantial evidence, have obviated one objection, however irrational, to the new inoculation, viz. on account of the assigned origin of the Cow-pock being this disease of horses.

9. A clamour was raised against the inoculation of the Cow-pock, on account of its being asserted that a person was liable to the Cow-pock repeatedly, although it destroyed the susceptibility to the Small-pox; but numerous experiments have shown, that the same person is only susceptible, constitutionally, of the Cow-pock by inoculation once, as is the case with the Small-pox.

10. The introduction of the Cow-pock was also clamorously objected to, on account of its being an infectious disease, which could affect even persons who had already undergone the Small-pox. It was said, "there were diseases enough already, and no advantages from the Cow-pock could compensate for the addition to the number." Many experiments of inoculation with Cow-pock matter have shown, that those who have undergone the Small-pox cannot take the Cow-pock constitutionally.

---

* Of the numerous instances communicated to me of such eruptions, I shall mention a few, although I must acknowledge that but one case of this sort has occurred in my own practice these eight months. 1. In a person inoculated by Dr. Jenner, in the country, who came to town, and was under the care of Mr. Cotton, "the eruptions bore much resemblance to the inoculated Small-pox, in number from twelve to twenty." See Mr. Cotton's letter to Dr. Pearson. 2. No one has been more active and successful than Mr. Ring; this gentleman, whose experience I know has been very extensive, could not avoid eruptive cases, although he got matter with great pains from different sources. "I have," says Mr. Ring, in his letter to Dr. Pearson, "inoculated thirty patients with matter given to me by Mr. Patherus, and to him by Dr. Jenner. One of these had about 150 pustules; these were not distinguishable from variolous ones by any diagnostics with which I am acquainted. The matter was purulent; became perfectly opaque, and, on exsiccation, formed a scab as large as that left by the Small-pox." 3. No person's evidence on this point is better than Mr. Grose's, (see last number of the Medical and Physical Journal, and his letter to Dr. Pearson) "Eruptions similar to the Small-pox have appeared, though a new lancet had been used, &c." 4. See also Mr. Shorter's letter, Medical and Physical Journal for April, 1800, p. 349, for an eruptive case. 5. In the practice of inoculation for two months, at the *Vaccine Institution*, not a single eruptive case occurred; but, within this fortnight past, notwithstanding the most religious precautions by Messrs. Keate and Gunning, such a case occurred.

